# Supplementary material for: Chemical Characterization of Capsule-Brewed Espresso Coffee Aroma from the Most Widespread Italian Brands by HS-SPME/GC-MS
Source: Molecules. 2020 Mar 5;25(5):1166. doi: 10.3390/molecules25051166 (PMC7179241; doi:10.3390/molecules25051166)
Supplement: Supplementary file 1 [file molecules-25-01166-s001.zip › Table S1_Molecules-720172 (finalproof).docx]

|  | **PC3 SCORE COEFFICIENTS** | | |
| --- | --- | --- | --- |
| **Variables** | **1** | **2** | **3** |
| 2-methylpropanal | -0.03 | 0.00 | 0.01 |
| 2-methylbutanal | -0.04 | 0.02 | 0.02 |
| 3-methylbutanal | -0.05 | 0.00 | -0.04 |
| furfural | -0.27 | 0.47 | 0.16 |
| benzaldehyde | -0.05 | 0.02 | -0.05 |
| 5-methyl-2-furfural | -0.36 | 0.49 | -0.01 |
| 2-methybenzaldehyde | -0.04 | -0.03 | -0.01 |
| 2-methyl-3-2-furylacrolein | -0.08 | 0.00 | 0.10 |
| 1H-pyrrole-2-carboxaldehyde | -0.05 | -0.01 | 0.01 |
| methyl acetate | -0.02 | -0.01 | 0.01 |
| ethyl acetate | -0.01 | 0.01 | -0.01 |
| propanoic acid, 3-methyl-, methylester | -0.04 | 0.05 | 0.06 |
| methyl isovalerate | 0.00 | 0.00 | 0.00 |
| prenyl acetate | -0.01 | 0.02 | 0.01 |
| furfurylformate | -0.03 | 0.01 | 0.06 |
| 2-furfuryl-propanoate | -0.09 | -0.06 | -0.11 |
| 2-methylfuran | -0.02 | -0.01 | -0.01 |
| 2,5-dimethylfuran | 0.00 | 0.00 | 0.00 |
| vinylfuran | 0.00 | 0.00 | 0.00 |
| 2-methoxymethylfuran | -0.02 | 0.01 | 0.00 |
| 2-butylfuran derivative | -0.06 | 0.01 | 0.06 |
| furan,2,2'-methylenbis | -0.10 | -0.07 | -0.03 |
| difurfuryl ether | -0.14 | -0.23 | -0.11 |
| 2-butanone | -0.01 | 0.00 | 0.01 |
| 2-pentanone | -0.01 | 0.00 | 0.00 |
| 2,3-butandione | -0.01 | 0.01 | 0.01 |
| 2,3-pentanedione | -0.02 | 0.03 | 0.00 |
| 3-penten-2-one | 0.00 | 0.00 | 0.01 |
| 2,3-hexanedione | -0.01 | 0.01 | 0.02 |
| 1-furan2-ylethanone | -0.11 | 0.09 | 0.05 |
| 1-furan-2-ylbutan-2-one | -0.02 | 0.00 | -0.01 |
| 1-1-methyl-1H-pyrrol-2-yl ethanone | -0.09 | -0.05 | -0.13 |
| furfural acetone derivative | -0.02 | -0.01 | 0.01 |
| pyridine | -0.23 | -0.39 | 0.43 |
| 4(H)-pyridine, n-acetyl | -0.05 | -0.01 | 0.01 |
| pyrazine | -0.02 | 0.00 | 0.03 |
| 2-methyllpyrazine | -0.17 | 0.10 | 0.30 |
| 2,5-dimethylpyrazine | -0.10 | 0.06 | 0.11 |
| 2,6-dimethylpyrazine | -0.10 | 0.05 | 0.16 |
| 2-ethylpirazine | -0.07 | 0.04 | 0.07 |
| 2,3-dimethylpyrazine | -0.02 | 0.01 | 0.02 |
| 2-ethyl-6-methylpyrazine | -0.08 | 0.03 | -0.01 |
| 2-ethyl-5-methylpyrazine | -0.06 | 0.03 | -0.01 |
| 2,3,5-trimethylpyrazine | -0.10 | -0.02 | 0.00 |
| 2-ethyl-3-methylpyrazine | -0.01 | 0.00 | 0.00 |
| 2,6-diethylpyrazine | -0.04 | -0.01 | -0.02 |
| 3-ethyl-2,5-dimethylpyrazine | -0.12 | 0.01 | -0.05 |
| beta-linalool | -0.03 | 0.05 | 0.02 |
| p-menthene monoterpenoid derivative | -0.07 | -0.01 | -0.03 |
| p-menthene monoterpenoid derivative 2 | -0.08 | -0.04 | 0.00 |
| 1-methyl-1H-pyrrole | -0.02 | -0.03 | 0.00 |
| 1H-pyrrole derivative | -0.08 | -0.03 | 0.00 |
| 1-furfurylpyrrole | -0.19 | -0.11 | -0.19 |
| 2-acetyl-1H-pyrrole | -0.12 | -0.06 | 0.07 |
| 1-methyl-1H-pyrrole-2-carbaldehyde derivative | -0.11 | 0.04 | 0.02 |
| guaiacol 2-methoxyphenol | -0.10 | -0.15 | -0.01 |
| 2-methoxy-4-methylphenol | -0.08 | 0.05 | 0.02 |
| 4-ethylguaiacol | -0.07 | -0.16 | -0.09 |
| 2-methylphenol (o-cresol) | -0.03 | -0.02 | -0.01 |
| 3-methilphenol (m-cresol) | -0.02 | -0.02 | 0.01 |
| p-vinylguaiacol 2-methoxy-4-vinylvinylphenol | -0.10 | -0.08 | -0.15 |
| 2-methylsulfanylmethylfuran | -0.05 | -0.04 | -0.07 |
| unknown 1 | -0.23 | -0.39 | 0.43 |
| benzylalchool | -0.03 | -0.02 | -0.02 |
| 2-furanmethanol | -0.40 | 0.08 | 0.05 |
| 2-furanmethanolacetate | -0.48 | -0.20 | -0.55 |
| unknown 2 | -0.07 | 0.06 | 0.12 |
| uknown 3 | -0.02 | 0.00 | -0.01 |
| furfurylammine derivative | -0.06 | 0.00 | -0.01 |
| unknown 4 | -0.02 | -0.02 | -0.01 |
| indole | -0.01 | 0.00 | 0.00 |
| 3-methylindole | -0.01 | -0.03 | -0.01 |
