# Supplementary material for: Chemical Characterization of Capsule-Brewed Espresso Coffee Aroma from the Most Widespread Italian Brands by HS-SPME/GC-MS
Source: Molecules. 2020 Mar 5;25(5):1166. doi: 10.3390/molecules25051166 (PMC7179241; doi:10.3390/molecules25051166)
Supplement: Supplementary file 1 [file molecules-25-01166-s001.zip › Caption_TableS1.docx]

**Table S1**: PC3 score coefficients of variables related to concentrations (µg/L) of detected volatiles in EC samples.
